# Paracrine Interleukin 6 Induces Cerebral Remodeling at Early Stages After Unilateral Common Carotid Artery Occlusion in Mice

**DOI:** 10.3389/fcvm.2021.805095

**Published:** 2022-01-27

**Authors:** Melanie T. C. Kuffner, Stefan P. Koch, Marieluise Kirchner, Susanne Mueller, Janet Lips, Jeehye An, Philipp Mertins, Ulrich Dirnagl, Matthias Endres, Philipp Boehm-Sturm, Christoph Harms, Christian J. Hoffmann

**Affiliations:** ^1^Klinik und Hochschulambulanz für Neurologie mit Experimenteller Neurologie, Charité-Universitätsmedizin Berlin, Freie Universität Berlin, Humboldt-Universität zu Berlin, Berlin Institute of Health, Berlin, Germany; ^2^Center for Stroke Research Berlin, Charité-Universitätsmedizin Berlin, Berlin, Germany; ^3^NeuroCure Cluster of Excellence and Charité Core Facility 7T Experimental MRIs, Charité-Universitätsmedizin Berlin, Berlin, Germany; ^4^Core Unit Proteomics, Berlin Institute of Health at Charité- Universitätsmedizin Berlin, Max Delbrück Center for Molecular Medicine, Berlin, Germany; ^5^German Center for Cardiovascular Research (DZHK), Partner Site Berlin, Berlin, Germany; ^6^NeuroCure Clinical Research Center, Charité-Universitätsmedizin Berlin, Berlin, Germany; ^7^German Center for Neurodegenerative Diseases (DZNE), Partner Site Berlin, Berlin, Germany; ^8^Einstein Center for Neuroscience, Berlin, Germany; ^9^QUEST Quality, Ethics, Open Science, Translation, Center for Transforming Biomedical Research, Berlin Institute of Health, Berlin, Germany

**Keywords:** vascular disease, basic science research, proteomics, inflammation, carotid artery occlusion

## Abstract

**Aims:**

Carotid artery disease is frequent and can result in chronic modest hypoperfusion of the brain. If no transient ischemic attack or stroke occur, it is classified asymptomatic. In the long-term, though, it can lead to cognitive impairment. Fostering cerebral remodeling after carotid artery occlusion might be a new concept of treatment. Paracrine Interleukin 6 (IL-6) can induce such remodeling processes at early stages. However, it has neurodegenerative long-term effects. With this exploratory study, we investigated the effect of paracrine IL-6 on cerebral remodeling in early stages after asymptomatic carotid artery occlusion to identify new treatment targets.

**Methods and Results:**

To mimic a human asymptomatic carotid artery disease, we used a mouse model of unilateral common carotid artery (CCA) occlusion. We developed a mouse model for inducible paracrine cerebral IL-6 expression (Cx30-Cre-ERT2;FLEX-IL6) and induced IL-6 2 days after CCA occlusion. We studied the effects of paracrine IL-6 after CCA occlusion on neuronal connectivity using diffusion tensor imaging and on local proteome regulations of the hypo-perfused striatum and contralateral motor cortex using mass spectrometry of laser capture micro-dissected tissues. Paracrine IL-6 induced cerebral remodeling leading to increased inter-hemispheric connectivity and changes in motor system connectivity. We identified changes in local protein abundance which might have adverse effects on functional outcome such as upregulation of Synuclein gamma (Sncg) or downregulation of Proline Dehydrogenase 1 (Prodh). However, we also identified changes in local protein abundance having potentially beneficial effects such as upregulation of Caprin1 or downregulation of GABA transporter 1 (Gat1).

**Conclusions:**

Paracrine cerebral IL-6 at early stages induces changes in motor system connectivity and the proteome after asymptomatic CCA occlusion. Our results may help to distinguish unfavorable from beneficial IL-6 dependent protein regulations. Focusing on these targets might generate new treatments to improve long-term outcome in patients with carotid artery disease.

## Introduction

Carotid artery disease is highly prevalent in an aged population (1). It may lead to carotid artery stenosis or occlusion, causing chronic hypoperfusion of the brain and increasing the risk for cerebral infarction (2). In the long-term, carotid artery disease can cause white matter damage. When no transient ischemic attack or stroke occur it is classified asymptomatic. However, about 50% of these patients with asymptomatic carotid stenosis show signs of neuropsychological deficits such as reduced processing speed and learning (3).

Fostering cerebral remodeling after carotid artery occlusion might be a new concept of treatment to improve the long-term outcome. We have shown that the cytokine interleukin 6 (IL-6) is essential for post-ischemic angiogenesis and improves the outcome after stroke (4). Down-stream signaling of IL-6 in endothelial cells is needed to induce changes in the extracellular matrix and the molecular micro-environment fostering neuroplasticity and functional recovery (5). It has been shown that IL-6 improves repair after trauma (6). However, long-term elevated IL-6 is associated with cognitive decline (7). Indeed, the effects of IL-6 are complex and depend on its temporal (acute vs. long-term) and spatial (paracrine vs. systemic) expression.

We hypothesize that—comparable to its effects after stroke—paracrine IL-6 might induce beneficial remodeling processes in the acute phase after modest cerebral hypoperfusion induced by carotid artery disease while it might have detrimental effects in the long-term. Therefore, the aim of this exploratory study was to analyze early-stage cerebral remodeling mechanisms of paracrine brain-specific IL-6 within the first 3 weeks of modest chronic cerebral hypoperfusion. We used the mouse model of unilateral common carotid artery (CCA) occlusion to mimic an asymptomatic carotid artery stenosis in humans. In this mouse model, long-lasting modest hypoperfusion of the brain has been verified without the development of strokes or motor function deficits (8, 9). Comparable to humans, unilateral CCA stenosis induced neuropsychological deficits in mice primarily affecting learning. A prolonged increase of cerebral astrocytic IL-6 has been shown to cause neuroinflammation and neurodegeneration within a period of 3–24 month using a constitutive GFAP-IL6 mouse model and this effect depended on the heights of IL-6 expression (10–12). We developed a genetically modified mouse model for inducible brain-specific paracrine secretion of IL-6 to avoid this biasing effect before CCA occlusion. Using this mouse model, we studied the effects of a slight paracrine IL-6 increase on connectivity changes and local proteome changes.

With this study, we show that paracrine IL-6 induces adaptations in the motor system connectivity. In the proteome, we could differentiate proteins that might improve functional and cognitive outcome from proteins with unfavorable effects. By this, our results might help to identify molecular targets for new treatments of asymptomatic CCA occlusion.

## Methods

### Animals

All experimental procedures were approved by the local authorities (Landesamt für Gesundheit und Soziales, LaGeSo), Berlin (Reg G0119/16) and were conducted following the German animal protection law and local animal welfare guidelines. Mice were group-housed with *ad libitum* access to water. Food was restricted as a motivator for functional testing. Housing conditions and details are described in Supplementary Material.

We genetically engineered a mouse model by inducing a flip and excision (FLEX) cassette into the ROSA26 locus using recombinase-mediated cassette exchange containing an inverted IL-6 followed by a T2A self-cleavage sequence and mKate2. IL-6 was fused to a C-terminal myc-tag (EQKLISEEDL) via a spacer (GGSGGTGGS). Mice were crossbred with astrocyte-specific Cx30-Cre-ERT2 mice. The mice are bred locally on a C57BL/6J background and were backcrossed for 10 generations. We used mice for experiments that were hemizygous for Cx30-Cre-ERT2 and hemizygous for FLEX-IL-6. Mice that were hemizygous for FLEX-IL-6 served as control animals. The mice were at an age of 10–12 weeks. In total, 66 mice were used for the experiments. Of these mice, 64 mice were analyzed according to the preset exclusion criteria (see methods to prevent bias and exclusion criteria), and further 2 mice were excluded for technical reasons as specified in the section Results. Mouse groups were age- and sex-matched for experimental and control groups.

For surgery, mice were anesthetized with 1.5–3.5% Isoflurane and maintained in 1.0–2.5% Isoflurane with ~75/25 N_2_O/O_2_ and for pain relief, bupivacain gel (1%) was topically applied to the wound. For MRI, anesthesia was induced with 2.5% and maintained with 2.0–1.5% isoflurane (Forene, Abbot, Wiesbaden, Germany) delivered in a O_2_/N_2_O mixture (0.3/0.7 l/min) via a facemask under constant ventilation monitoring (Small Animal Monitoring & Gating System, SA Instruments, Stony Brook, New York, USA). Mice were euthanized in deep anesthesia with 100 μl Ketamine/Xylazine [0.7% Ketamine (10%, cb pharma), 0.8% Xylazine (20 mg/ml Xylavet, cb pharma)] per 10 g body weight given i.p. and cardially perfused with 0.9% physiological NaCl solution.

### Western Blot and Enzyme-Linked Immunosorbent Assay

IL-6 was semi-quantitatively analyzed by ELISA in serum samples and deep-frozen mouse brain sections as described in the Supplementary Material.

### Cell Culture, Transfection, and Real-Time RT-PCR

HEK 293T cells were transfected with plasmids coding for FLEX-IL-6, Cre-GFP fusion or both. Brain microvascular endothelial cells were treated with conditioned medium of these cultures for 1 h prior quantification of IL-6 mRNA and a reference transcript as described in detail in Supplementary Material.

### Mouse Surgery and Tamoxifen Injection

Mice were subjected to unilateral ligation of the left common carotid artery. Following the 3R principle of animal experiment reduction, mice with unilateral CCA occlusion served additionally as sham control for a transient occlusion of the middle cerebral artery (MCAO). In brief, surgery was performed analogously to the sham operation as established in our lab and described in detail in Supplementary Material, and tamoxifen was injected intraperitoneal on day two after surgery as a single dose or on three consecutive days (1 mg tamoxifen; Sigma, 10 mg/ml in 1:10 ethanol/corn oil).

### Behavioral Tests

Sickness behavior was assessed by modified DeSimoni's neuroscore at days 2, 7, 14, and 21 following unilateral CCA occlusion. The maximum score is 43 points, where a higher score indicates more deficits. Rotarod test was performed with three runs per trial with a recovery time of 30 min between trials. Shown is the average performance. Tests were performed on days 2, 7, and 14 after unilateral CCAo. Staircase skilled pellet reaching test was performed daily for 21 days of training before and up to 21 days after ligation of the CCA for conditioning as described previously (13). Outcome was measured as relative performance compared to the individual performance before surgery for each forepaw.

### Magnetic Resonance Imaging

Mice were examined 24 h after the onset of CCA using T2-weighted imaging. After 21 days, T2-weighted and diffusion tensor imaging (DTI) were performed as outlined in detail in Supplementary Material. Briefly, a custom brain atlas with 308 anatomical regions (154 right/154 left hemisphere) derived from the Allen mouse brain atlas was registered to the T2-weighted and diffusion MR images using ANTx2 (https://github.com/ChariteExpMri/antx2). DTI can measure the directionality of water diffusion in tissue, which is a proxy of the orientation of nerve fiber tracts in the respective voxel. Using computational models that take into account the orientation of water diffusion in all voxels in gray and white matter, long range fiber connections from one atlas region to another region can therefore be reconstructed. The number of reconstructed tracts is a measure of connectivity strength. From the DTI scan, a matrix of all possible combinations of those connections and their strength (termed DTI connectome) for each animal was calculated in mrtrix (https://www.mrtrix.org, shell scripts available at https://github.com/ChariteExpMri/rodentDtiConnectomics). Between group comparisons were carried out on the number of reconstructed streamlines between pairs of regions.

### Proteomic Analysis of Laser Capture Microscopy Samples by Mass Spectrometry

Briefly, left hemispheric ipsilateral fiber tracts in the striatum or the contralateral motor cortex were laser-captured 5 days after CCA occlusion and 3 days after i.p. injection of tamoxifen from frozen tissue sections and subjected to mass spectrometry and analysis as described in Jochner et al. (14) and in detail in Supplementary Material.

### Histology and Imaging Analysis

Briefly, mice were euthanized in deep anesthesia, blood was collected from the Vena cava after abdominal incision, and mice were cardially perfused with 0.9% physiological NaCl solution. For LCM/proteomics, protease inhibitor was added (1 tablet/50 ml saline solution) (cOmplete™, Roche). Brains were removed and snap-frozen in 2-methyl butane at −45°C using dry ice. Histological staining, imaging analysis, and antibodies used are described in Supplementary Material.

### Methods to Prevent Bias and Exclusion Criteria

Reporting conforms to the ARRIVE guidelines. Mice were excluded determined by a priori criteria if they had a visible stroke in MRI imaging 24 h after surgery (2 mice, 1 per genotype, respectively). Experimenters were blinded during the behavioral assessment, tissue processing, and data analysis. We observed no mortality. All other mice were included. Two samples after microdissection did not result in enough protein for analysis and were not further processed.

### Statistical Analyses

All data are presented as scatter dot plots with the mean ± standard deviation. Data were analyzed with GraphPad Prism version 8.2.0. A detailed description of the corresponding statistical analysis is provided in the figure legends or the respective proteomics and connectivity analysis methods.

## Results

### Development of the Cx30-Cre-ERT2;FLEX-IL6 Mouse Model for Paracrine Cerebral IL-6 Expression

**Figure 1:**
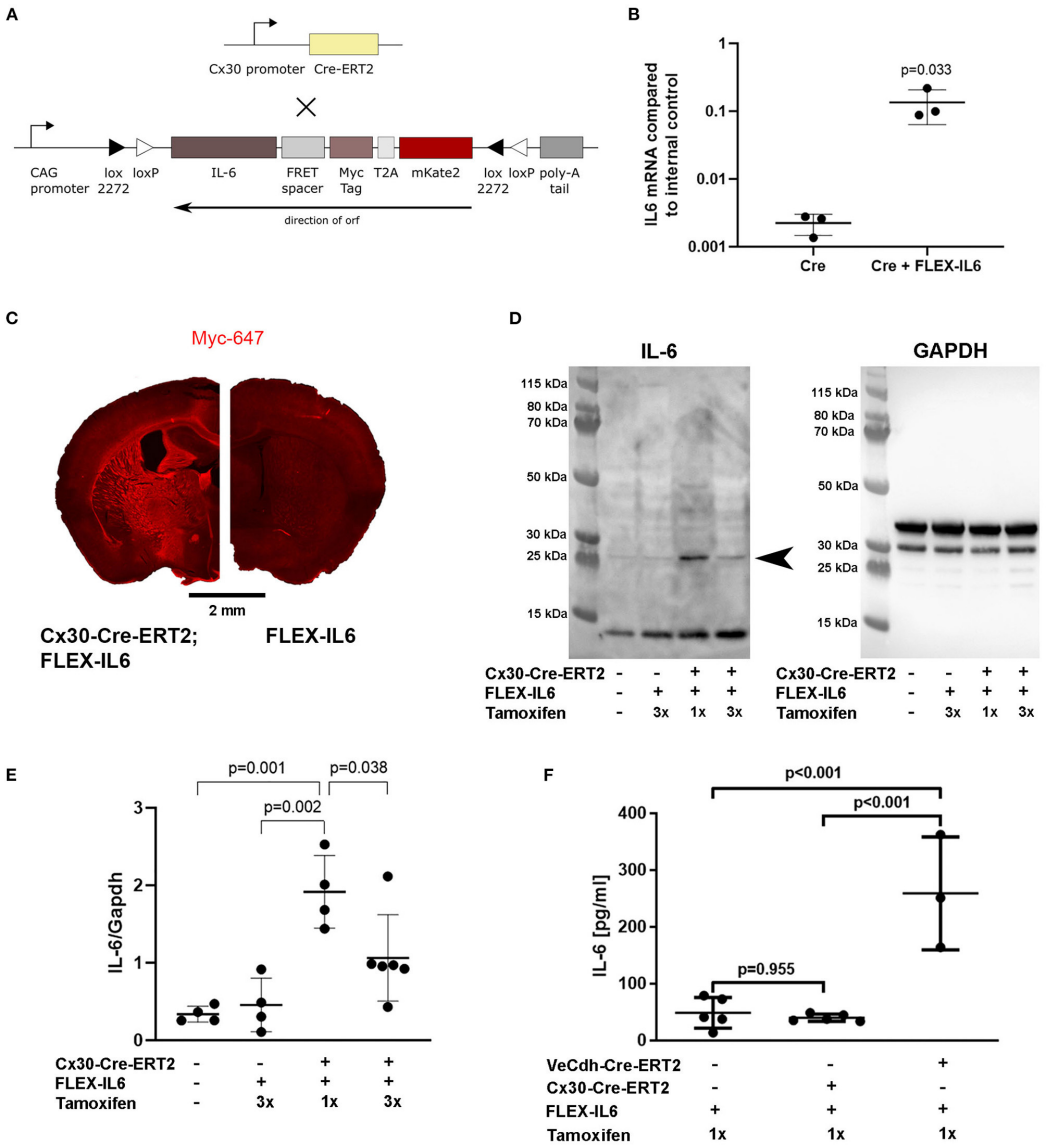
Development of the Cx30-Cre-ERT2;FLEX-IL6 mouse model for brain-specific IL-6 expression. **(A)** Design of the custom-made mouse line for inducible and traceable secretion of IL-6 (FLEX-IL6). The murine *Il6* sequence is linked to a myc-tag followed by the self-cleavage site T2A and mKate2. This construct is inverted and embedded in a flip and excision (FLEX) cassette in the silent R26 locus. To enable paracrine cerebral IL-6 secretion, we crossbred FLEX-IL6 mice with the astrocyte-specific inducible Cre-driver mouse line Cx30-Cre-ERT2. Upon tamoxifen administration, active Cre flips and excises one recombination site and thus permanently induces *Il6* expression in astrocytes. **(B)** Endothelial bEnd.3 were incubated for 1 h with conditioned cell culture supernatants of HEK cells transfected with Cre and the FLEX-IL6 construct or Cre only as negative control and *Il6* mRNA was determined by real-time RT-PCR. Ectopic IL-6 increases bEnd.3 IL-6 expression by almost 100x. *n* = 3 independent experiments. **(C)** Representative Myc-tag staining after tamoxifen administration. Upon induction with tamoxifen, myc-IL-6 is widely expressed in Cx30-CRE-ERT2;FLEX-IL6 mice, while there is no induction in FLEX-IL6 control mice. **(D,E)** Cerebral IL-6 protein levels were determined by immunoblot analysis of brain homogenates of Cx30-Cre-ERT2;FLEX-IL6 mice receiving one mg tamoxifen for 1 or 3 days from *n* = 4–6 mice per group as displayed in the scatter dot plots. FLEX-IL6 (*n* = 4) mice receiving 1 mg tamoxifen for 3 days and wild-type C57BL/6N (*n* = 4) served as negative controls. Immunoblot IL-6 intensities were normalized to GAPDH intensity (~37 kD). There is a moderate increase of IL-6 in Cx30-Cre-ERT2;FLEX-IL6 mice (*n* = 4) receiving a single tamoxifen dose compared to the control group (~25 kD, arrowhead closed). Mice that received three doses of tamoxifen (*n* = 6) showed increased but significantly lower brain IL-6 than after a single dose. **(F)** IL-6 serum levels were determined using ELISA measurement of mice with astrocytic IL-6 expression (Cx30-Cre-ERT2;FLEX-IL6, *n* = 5), mice with endothelial IL-6 expression (VeCdh-Cre-ERT2; FLEX-IL6, *n* = 37), and Cre-negative controls (*n* = 5). There was a marked increase in serum IL-6 upon endothelial expression. At the same time, IL-6 remained at baseline level comparable to Cre-negative controls upon astrocytic expression. Graphs show scatter dot plots of data from independent samples ± standard deviations. **(B)** Two-tailed unpaired Student's *t*-test. **(E,F)** One-way ANOVA with Tukey's multiple comparison test.

To evaluate the effects of paracrine cerebral IL-6 on cerebral remodeling mechanisms after unilateral CCA occlusion, we created a custom-made mouse model for brain-specific IL-6 secretion at a deliberately chosen time-point. In this model, a FLEX cassette is integrated into the R26-locus. The FLEX cassette contains murine IL-6 connected to a myc-tag via a linker and mKate2 via a T2A self-cleavage site (Figure 1A). This myc-tagged IL-6 was bioactive, which can be seen by the induction of IL-6 in endothelial cells *in vitro* (Figure 1B). We have previously shown that IL-6 induces the production of IL-6 in endothelial cells by a positive feedback loop (4). Crossbreeding of FLEX-IL6 mice with Cx30-Cre-ERT2 mice resulted in the Cx30-Cre-ERT2;FLEX-IL6 mouse model for tamoxifen dependent inducible activation of IL-6 secretion restricted to astrocytes and by this restricted to the brain. Upon tamoxifen administration, we observed a widespread IL-6 expression in the Cx30-Cre-ERT2;FLEX-IL6 mice using immunofluorescence staining for the myc-tag, which is fused to the ectopic IL-6. This expression was not present in the FLEX-IL6 control mice (Figure 1C). IL-6 immunoblots of brain homogenate showed a tamoxifen-dependent increase of IL-6 locally in the brain 5 days after a single administration of tamoxifen (Figures 1D,E). However, after higher tamoxifen dosing with administration on three consecutive days, we observed decreased IL-6 compared to 1-day administration. In FLEX-IL6 mice without Cx30-Cre-ERT2 we observed no induction of IL-6 compared to wild-type mice. Therefore, we used a single dose of tamoxifen for IL-6 induction in the following experiments. FLEX-IL6 mice without Cre but with tamoxifen treatment served as negative controls. To control for brain specificity of IL-6 induction in the setting of a CCA occlusion, we determined the serum IL-6 levels after tamoxifen administration after surgery (Figure 1F). We identified no increase of systemic serum IL-6 levels in Cx30-Cre-ERT2;FLEX-IL6 mice while there was a substantial increase of serum IL6-levels when using an endothelial Cre-driver mouse model (VeCdh-Cre-ERT2;FLEX-IL6). Therefore, using the Cx30-Cre-ERT2;FLEX-IL6 mouse model we can induce paracrine cerebral IL-6 without affecting systemic IL-6 levels.

### Paracrine IL-6 Production Does Not Induce Ischemic Tissue Damage or Worsen Functional Outcome After Unilateral CCA Occlusion

**Figure 2:**
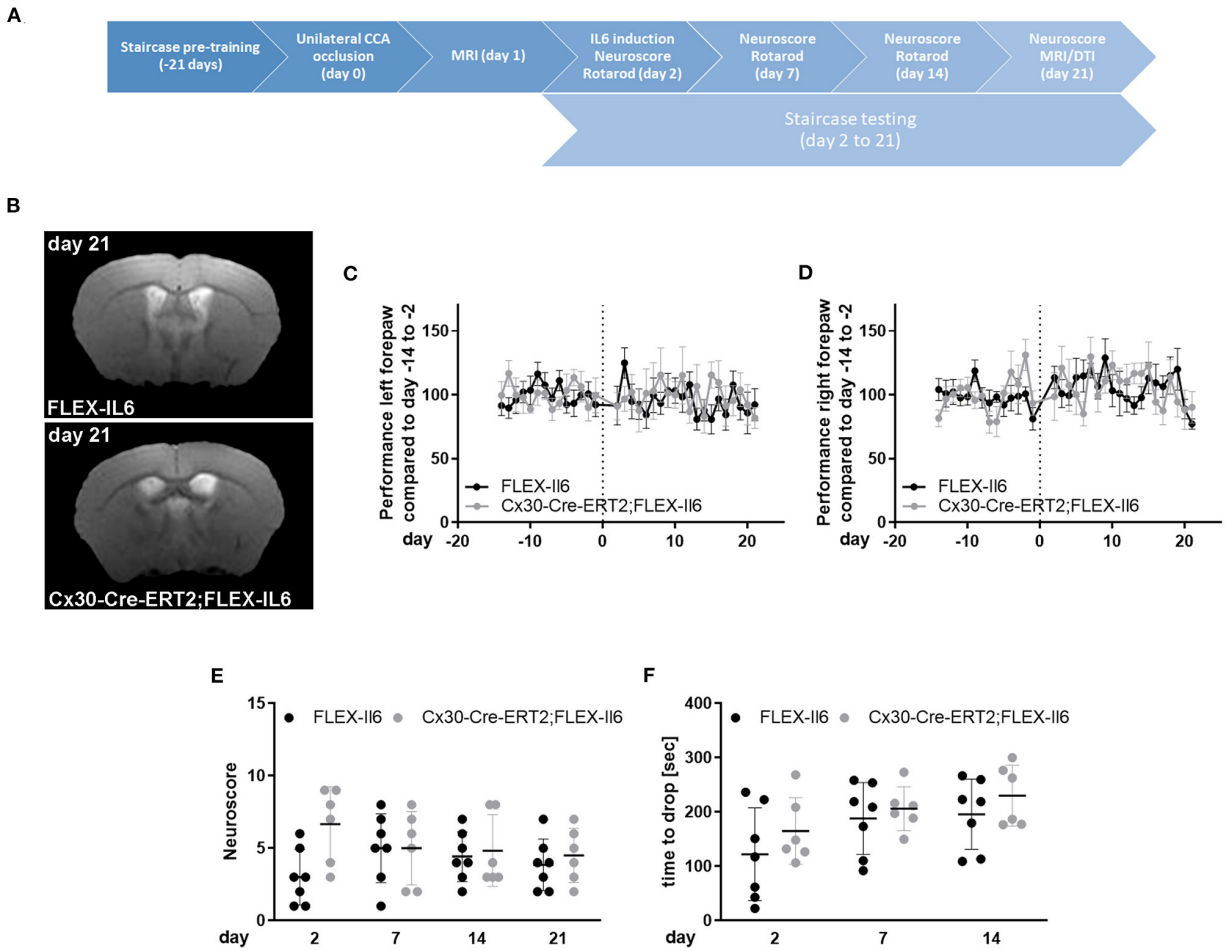
Paracrine IL-6 does not worsen functional outcome or induce strokes after unilateral asymptomatic CCA occlusion. **(A)** Time-scale: 8 Cx30-Cre-ERT2;FLEX-IL6 and 7 FLEX-IL6 mice were pre-trained in the staircase test for 21 days. On day 0, mice received a left-sided unilateral CCA occlusion and an MRI on day 1 after surgery. One Cx30-Cre-ERT2;FLEX-IL6 and one FLEX-IL6 was excluded due to developing a stroke immediately after surgery. On day 2 after surgery, paracrine IL6 was induced by a single tamoxifen administration. Motor function was evaluated on day 2, day 7, and 14 with the Rotarod test and day 21 with the DeSimoni Neuroscore. Forepaw function was evaluated daily with the staircase test from day 2 to day 21. **(B)** T2-weighted MRI scans showed no ischemic lesion or white matter damage 21 days after unilateral carotid occlusion in both genotypes. **(C,D)** Staircase test for the left **(C)** and right **(D)** forepaw. The performance was normalized to the individual performance before surgery. No change in performance was observed compared to the performance before surgery and between genotypes. **(E)** Overall sickness and neurological status were determined by DeSimoni Neuroscore. Cx30-Cre-ERT2;FLEX-IL6 mice (*n* = 6) showed a transient increase in sickness behavior immediately after IL6 induction, which reached no significance compared to FLEX-IL6 control mice (*n* = 7). **(F)** Gross motor performance was evaluated using the RotaRod test. After the intervention, the average time to drop on the moving Rotarod increases over time in the experimental and the control group. There is no significant difference between groups. Graphs show scatter dot plots of data from individual mice ± standard deviations. **(C–F)** Two-way ANOVA with Sidak's multiple comparison test.

We used the mouse model of unilateral CCA occlusion for studying the effects of modest cerebral hypoperfusion to mimic human asymptomatic carotid artery disease. In this mouse model, no deficits in sensorimotor functions occur. We excluded mice (one Cx30-Cre-ERT2;FLEX-IL6 and one FLEX-IL6) that developed a stroke peri-interventional. Figure 2A shows the time-scale of the experimental setup. We verified that mice did not develop an ischemic lesion in the long term using MR-imaging on day 21 after surgery (Figure 2B). Additionally, we assessed sickness behavior and motor skills using behavioral testing in the 21-day follow-up period after CCA occlusion. Skilled pellet retrieval off a staircase gives a readout for front paw fine motor skills. We observed no effect on fine motor functions of the forepaw in the wild-type and experimental group (Figures 2C,D). In both groups and for both front paws, the performance was consistently similar to the average baseline performance before unilateral CCA occlusion. The overall sickness and neurological performance (DeSimoni neuroscore) did not differ significantly between groups and over time (Figure 2E). Gross motor skill assessed by RotaRod showed no impairment of motor function (Figure 2F). The average time to drop did not differ significantly for individual days in-between groups. Therefore, elevated paracrine cerebral IL-6 had no detectable adverse effect on motor functional outcome or general sickness in a model of asymptomatic CCA occlusion.

### Paracrine IL-6 Is a Driver of Neuronal Network Remodeling After Unilateral CCA Occlusion

**Figure 3:**
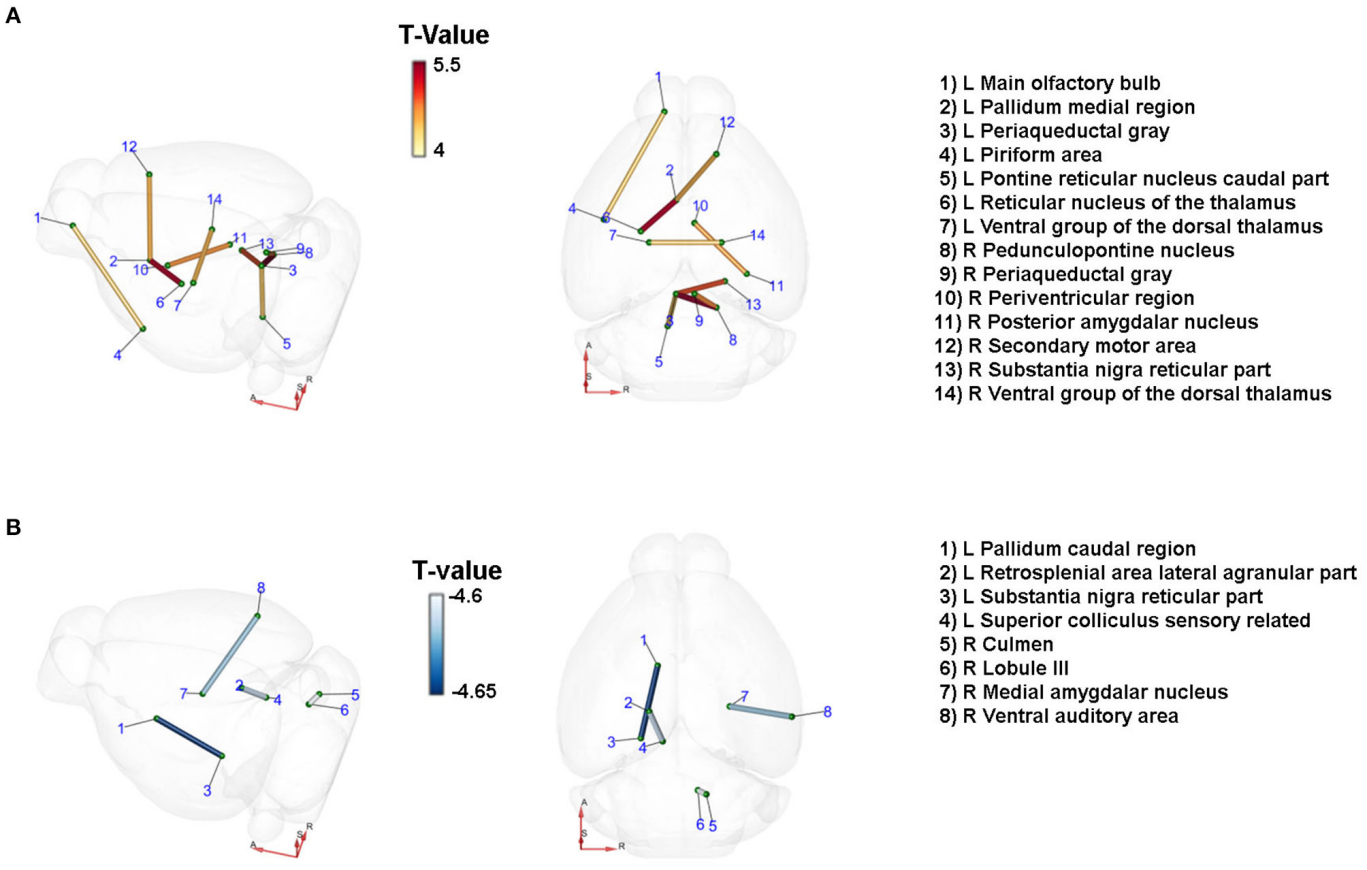
Paracrine IL-6 is a driver of network remodeling after unilateral CCA occlusion. Connections with changes in diffusivity comparing the astrocytic IL-6 (*n* =6) to control animals (*n* = 7) at d21 after left CCA occlusion. Coordinates: A = anterior, R = right, S = superior. T-statistics were applied, and all values with *p* < 0.001 were chosen as relevant. Connections were verified by cross-checking to the Janelia Neuron Browser or Allen Brain Mouse Connectivity Explorer. **(A)** Nine connections showed an increase in connectivity. Of those, four were inter-hemispheric connections. Especially connections to the ipsilateral periaqueductal gray increased along with the axis from the contralateral secondary motor cortex to the ipsilateral pallidum and subsequently the thalamus. **(B)** Four connections showed decreased connectivity. On the ipsilateral side, the caudal pallidum and the reticular part of the substantia nigra as well as the connection between the retrosplenial area and the superior colliculus were reduced in strength. On the contralateral side, the connections between the culmen and lobule III and between the medial amygdalar nucleus and the ventral auditory area were decreased.

We studied the changes in the brain's connectome by DTI 21 days after unilateral CCA occlusion in an unbiased exploratory approach using an atlas with 154 anatomical regions per hemisphere, resulting in 23,562 [(154^*^154) – 154] possible connections. Paracrine IL-6 production induced a significant change in connectivity in the context of CCA occlusion (Figures 3A,B). At a significance threshold of *p* < 0.001, 17 connections were significantly altered. The physiological feasibility of the connections was verified by cross-checking in the Allen brain connectivity atlas and Janelia Neuron Browser. In total, 3 out of 17 connections could not be verified and were therefore excluded. There was a gain in connectivity between 4 inter-hemispheric connections. In the hypoperfused hemisphere, 3 connections were strengthened, and in the contralateral hemisphere 2 connections. All 4 connections with lowered connectivity were intrahemispheric, with 2 connections reduced on each side. Qualitative assessment of connectivity hints at an overall gain in inter-hemispheric connectivity and, thus, a potential compensation by the contralateral non-affected hemisphere, though unilateral CCA occlusion was functionally asymptomatic.

In the brainstem, the ipsilateral periaqueductal gray showed increased connectivity to several other nuclei of the ipsi- and contralateral brainstem. We identified increased connectivity of the periaqueductal gray to the ipsilateral caudal part of the pontine reticular nucleus, the pedunculopontine nucleus, and the contralateral substantia nigra, and the contralateral periaqueductal gray. The ipsilateral pallidum, part of the basal ganglia, had strengthened connectivity to the ipsilateral reticular nucleus of the thalamus and the contralateral secondary motor area. The connectivity between the ipsi- and the contralateral ventral group of the dorsal thalamus—the relay between the basal ganglia and the primary motor cortex—increased. There was a decrease in connection strength to the ipsilateral reticular substantia nigra. Notably, we identified mainly changes in connections related to motor functions.

### Paracrine IL-6 Induces Changes in Protein Composition Within the Striatum

**Figure 4:**
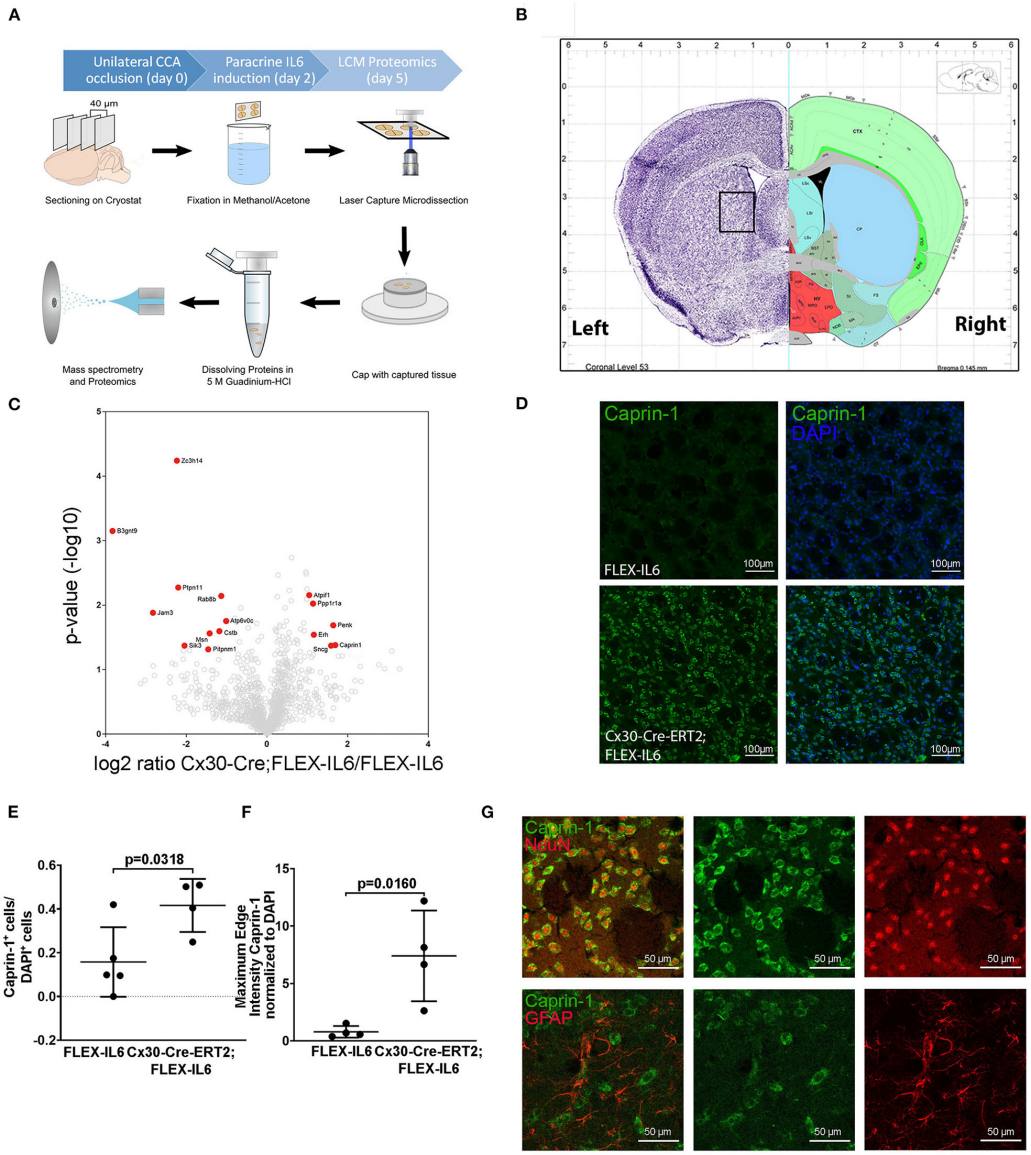
Paracrine IL−6 induces changes of the proteome in the striatum. **(A)** Schematic illustration of the laser capture microscopy (LCM) workflow. Five FLEX-IL6 and four Cx30-Cre-ERT2;FLEX-IL6 mice were used. Paracrine IL-6 secretion was induced 2 days after left-sided unilateral CCA occlusion. Brain tissue is sectioned into 40 μm thick coronal slices and mounted on PEN membrane slides. After methanol/acetone fixation, the tissue is processed with the laser capture microscope. Striatal fiber bundles on the hypoperfused side were isolated. The addition of 5 M guanidinium chloride dissolves the tissue and denatures the proteins. The sample was analyzed using mass spectrometry for proteome analysis. **(B)** Illustration (Allen mouse Brain Atlas) of the anatomical region used for LCM. **(C)** The volcano plot shows log ratio and *p*-value distribution of all quantified proteins. Proteins with significantly (*p* < 0.05, fold change >/ <2) altered expression are labeled in red. We identified 16 differentially regulated proteins listed in Table 1. **(D)** Comparison of caprin-1 staining in FLEX-IL6 and Cx30-Cre-ERT2;FLEX-IL6 mice confirms elevated caprin-1 expression in mice with increased paracrine IL-6. **(E,F)** Automated quantification of nuclei and co-detection of Caprin-1 intensity resulted in a significant increase of the edge nuclei intensity in the caprin−1 channel normalized to DAPI intensity. **(G)** Co-staining of caprin-1 and NeuN and GFAP showed that caprin-1 is localized exclusively in neurons and not detected in astrocytes. Graphs show scatter dot plots of data from individual mice ± standard deviations. **(E,F)** Two-tailed unpaired Student's *t*-test.

To evaluate the molecular adaptive mechanisms of paracrine cerebral IL-6 after CCA occlusion, we analyzed the effect on protein composition within the ipsilateral striatum. This anatomical region is subject to reduced perfusion. As particularly white matter is damaged in chronic cerebral hypoperfusion, we chose to analyze the striatal fiber tracts, including the capsula interna and the corticospinal tract. To isolate those regions, we used laser capture microdissection (LCM). In our experiment, we assessed proteome changes in the ipsilateral fiber tracts within the hypoperfused striatum on day 5 after occlusion of the CCA for early-stage effects (Figures 4A,B). In total, we identified 2,075 proteins in the striatal samples with a 1% FDR threshold. Within these, 16 proteins showed IL-6 dependent differential abundance (Figure 4C; Table 1). Surprisingly, IL-6 induced no angiogenesis-related proteins in this anatomical region. Mostly proteins related to neuronal functions and stress response such as Synuclein gamma (Sncg), Proenkephalin (Penk), ATP Synthase Inhibitory Factor Subunit 1 (Atpif1), Protein Phosphatase 1 Regulatory Inhibitor Subunit 1A (PP1R1a), and Caprin-1 were induced. STRING GO term analysis of downregulated proteins revealed that most downregulated proteins are membrane-bound organelle and Golgi apparatus related (Cstb, Atp6v0c, Jam3, Rab8b, Pitpnm1, Ptpn11, Zc3h14, Msn, Sik3, B3gnt9). We exemplarily confirmed the induction of Caprin-1 in the paracrine IL-6 expressing mice with immunofluorescence staining (Figures 4D–F). Caprin-1 was found in neurons of the striatum, and not in astrocytes (Figure 4G).

**Table 1:** IL-6 dependent protein regulations in the striatal fiber tracts^*^.

**UNIPROT ID**	**Protein names**	**Gene symbol**	**Difference LFQ log2 ratio IL-6/ controls**	***p*-value (–log 10)**	**Biological process relevant to cerebral functions (AmiGO 2 or Reference)**
**Upregulated proteins**					
Q60865	Caprin-1	*Caprin1*	1,689	1,380	Positive regulation of dendritic spine morphogenesis
P22005, B1AZQ0	Proenkephalin-A;	*Penk*	1,643	1,690	Locomotory behavior, behavioral fear response
Q9Z0F7	Gamma-synuclein ynuclein	*Sncg*	1,593	1,378	Regulation of dopamine secretion, negative regulation of neuronal death
P84089, G3UW85	Enhancer of rudimentary homolog	*Erh*	1,163	1,542	–
Q9ERT9	Protein phosphatase 1 regulatory subunit 1A	*Ppp1r1a*	1,143	2,028	Promotes memory and learning (15)
E9PV44, O35143	ATPase inhibitor, mitochondrial	*Atpif1*	1,050	2,153	Regulation of ATP metabolic process (16)
**Downregulated proteins**					
E9Q9C5, P63082, D3Z3B2	V-type proton ATPase 16 kDa Proteolipid subunit	*Atp6v0c*	−1,019	1,758	–
P61028	Ras-related protein Rab-8B	*Rab8b*	−1,139	2,144	–
Q62426	Cystatin-B	*Cstb*	−1,185	1,598	Locomotory behavior, frameshift mutation leads to neurodegeneration (17)
P26041	Moesin	*Msn*	−1,419	1,562	Long-term memory (18)
P35235	Membrane-associated phosphatidylinositol transfer protein 1	*Pitpnm1*	−1,448	1,316	Brain development
E9PU87, F6U8X4, F6U6U5	Serine/threonine-protein kinase SIK3	*Sik3*	−2,043	1,373	–
P35235	Tyrosine-protein phosphatase non-receptor type 11	*Ptpn11*	−2,203	2,271	Axon guidance, brain development, cerebellar cortex formation
Q8BJ05	Zinc finger CCCH domain-containing protein 14	*Zc3h14*	−2,233	4,239	Knockdown reduces Tau aggregation (19)
Q9D8B7	Junctional adhesion molecule C	*Jam3*	−2,828	1,881	Axon regulation, myelination, blood-brain-barrier disruption (20)
F8WI62, Q8VI16	UDP-GlcNAc:betaGal beta-1,3-N-acetylglucosaminyltransferase 9	*B3gnt9*	−3,829	3,153	–

* *xUp- and down-regulated proteins in striatal corticospinal tract samples ipsilateral to the CCAo. The proteins are sorted by their expression change indicated by the difference of the LFQ log2 ratio between IL-6 animals and controls. Significance is indicated by a log10 p-value*.

### Paracrine IL-6 Induces Changes in Protein Composition Within the Contralateral Motor Cortex

**Figure 5:**
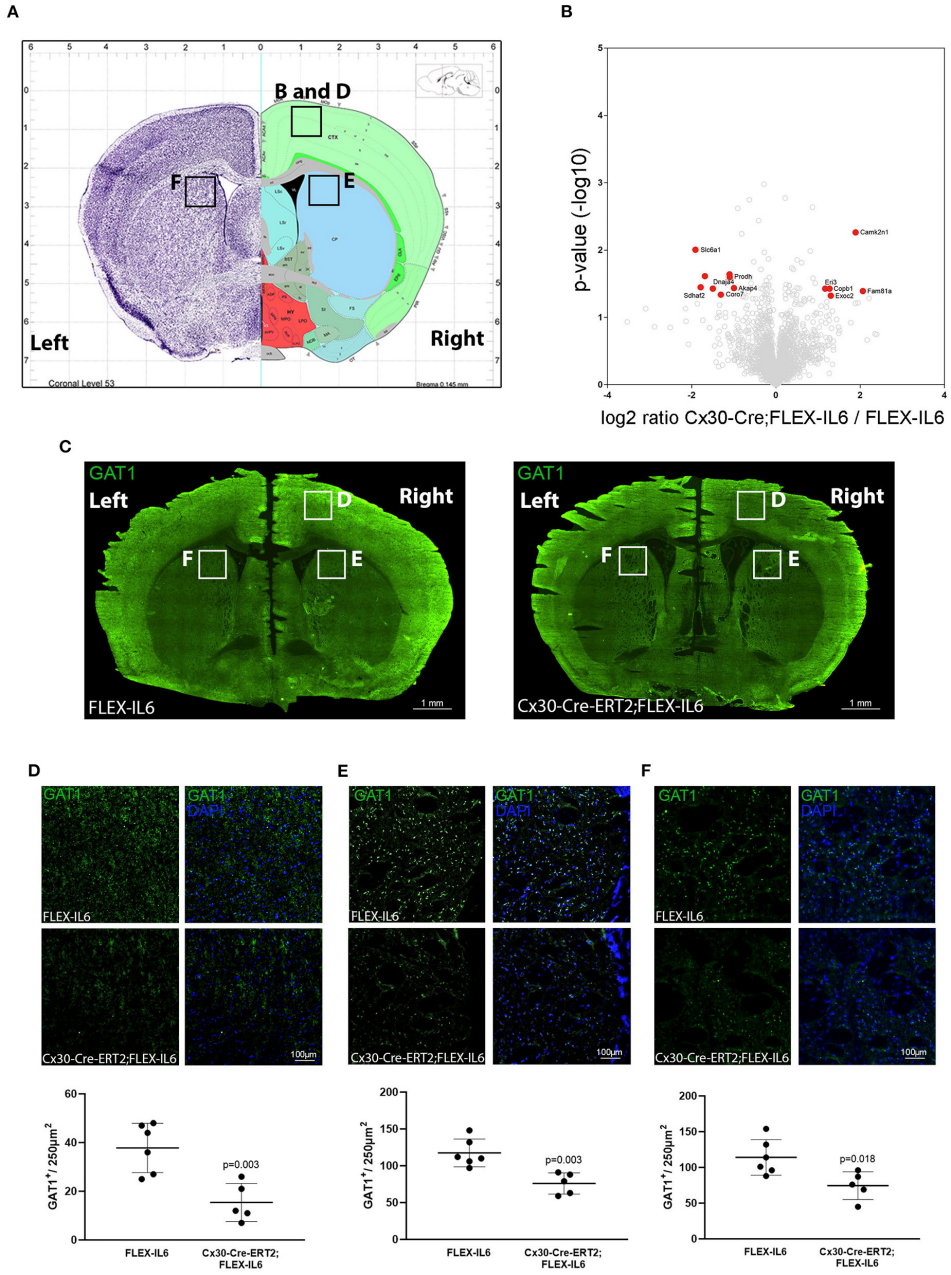
Paracrine IL−6 induces changes of the proteome in the contralateral motor cortex. **(A)** Location of the dissected tissue and histological analysis (Allen mouse Brain Atlas). A part of the motor cortex was isolated on the contralateral hemisphere and the proteome changes were analyzed. **(B)** The volcano plot shows log ratio and *p*-value distribution of all quantified proteins. Proteins with significantly (*p* < 0.05, fold change >/ <2) altered expression are labeled in red. We identified 13 differentially regulated proteins listed in Table 2. **(C)** Representative multiple image alignment of Gat1 staining. **(D)** Gat1 staining and quantification in the contralateral motor cortex shows besides focal staining a diffuse signal. The number of focal Gat1 spots is reduced in Cx30-Cre-ERT2;FLEX-IL6 (FLEX-IL6 *n* = 6, Cx30-Cre-ERT2;FLEX-IL6 *n* = 5). In the contralateral **(E)** and ipsilateral **(F)** caudate putamen, Gat1 is strongly expressed with reduction of expression in Cx30-Cre-ERT2;FLEX-IL6 mice. Graphs show scatter dot plots of data from individual mice ± standard deviations. Two-tailed unpaired Student's *t*-test.

Analyzing the effects of paracrine IL-6 on the connectivity after CCA occlusion we identified increased connectivity of the contralateral motor cortex to the ipsilateral hypoperfused basal ganglia. We therefore evaluated the molecular changes in the contralateral motor cortex via LCM and subsequent proteome analysis (Figure 5A). We identified 2,578 proteins and 13 proteins in the cortical samples that showed differential abundance due to paracrine IL-6 (Figure 5B; Table 2). Here, STRING GO term analysis of altered proteins showed that high abundance proteins are almost all linked to the plasma membrane system or endomembrane system. Low abundance proteins are mostly part of protein complexes and membrane components (Coa3, Coro7, Dnaja4, Sdhaf2, Slc6a1, Vamp3) including Vamp3—a component of the SNARE complex—or mitochondrial proteins (Coa3, Dnaja4, Prodh, Sdhaf2). Amongst these, we exemplarily analyzed Slc6a1 (also known as Gat1) in histological stainings in a different set of mice than used for LCM-proteomics (Figure 5C). We confirmed Gat1 suppression in the contralateral motor cortex (Figure 5D). There was widespread suppression of Gat1 in the brain with a noticeable reduction of Gat1 in the caudate putamen in both hemispheres (Figures 5E,F).

**Table 2:** IL-6 dependent protein regulations in the contralateral motor cortex^*^.

**UNIPROT ID**	**Protein names**	**Gene symbol**	**Difference LFQ log2 ratio IL-6/ controls**	***p*-value (–log 10)**	**Biological process relevant to cerebral functions (AmiGO 2 or Reference)**
**Upregulated proteins**					
Q3UXZ6	Protein FAM81A	*Fam81a*	2,061	1,390	Postsynaptic density (21)
Q6QWF9, Q78WH7	Calcium/calmodulin-dependent protein kinase II inhibitor 1;	*Camk2n1*	1,890	2,266	Prevents long-term memory loss (22), impairs motor learning and LTP (23)
Q9D4H1	Exocyst complex component 2	*Exoc2*	1,301	1,319	Mutations cause brain developmental defects (24)
Q9JIF7, Q8C460	Coatomer subunit beta	*Copb1*	1,262	1,428	Part of COPI complex, reduces amyloid plaques (25)
**Downregulated proteins**					
A0A0R4J1R9, Q8C460	ERI1 exoribonuclease 3	*Eri3*	1,185	1,424	–
Q60662	A-kinase anchor protein 4	*Akap4*	−0,990	1,433	–
E9Q6W2, Q9D2R6	Cytochrome c oxidase assembly factor 3 homolog, mitochondrial	*Coa3*	−1,092	1,641	Mitochondrial cytochrome c oxidase assembly
A0A0R4J1T9, Q9WU79	Proline dehydrogenase 1, mitochondrial	*Prodh*	−1,136	1,606	Reduced activity causes cognitive impairment (26)
G3X9L5, Q9D2V7	Coronin; Coronin-7	*Coro7*	−1,334	1,343	Actin filament organization
Q9JMC3	Protein FAM81A	*Dnaja4*	−1,459	1,430	–
P63024	Vesicle-associated membrane protein 3	*Vamp3*	−1,695	1,615	SNARE complex, inhibition reduces microglia activation (27)
Q8C6I2	Succinate dehydrogenase assembly factor 2, mitochondrial	*Sdhaf2*	−1,815	1,449	Mitochondrial electron transport, succinate to ubiquinone
P31648	Sodium- and chloride-dependent GABA transporter 1	*Slc6a1*	−1,918	2,004	Gamma-aminobutyric acid import

* *Up- and down-regulated proteins in the motor cortex contralateral to the CCAo. The proteins are sorted by their expression change indicated by the difference of the LFQ log2 ratio between IL-6 animals and controls. Significance is indicated by a log10 p-value*.

## Discussion

In this study, we show for the first time that paracrine cerebral IL-6 induces cerebral remodeling at an early stage after asymptomatic unilateral carotid occlusion within the first 3 weeks leading to increased inter-hemispheric connectivity and changes in motor system connectivity. By studying the molecular mechanisms induced by paracrine IL-6 early after CCA occlusion, we identified proteins that might serve as targets for potential treatment strategies aimed at cerebral remodeling to improve long-term functional outcome after carotid occlusion while avoiding adverse effects.

We showed that paracrine IL-6 induces restructuring mechanisms of neuronal connectivity after unilateral CCA occlusion. The main alterations were seen in strengthened interhemispheric connections between the thalamus and a strengthened connection between the contralateral motor area to the ipsilateral pallidum and the thalamic reticular nucleus. Moreover, there were increases in several connections linked to the periaqueductal gray and the hindbrain in general. The periaqueductal gray is a component of motor systems as an interface between the forebrain and the lower brainstem (28). We assume that this finding represents a general strengthening of downstream motor signaling toward the spinal cord.

Proteome analysis of the hypoperfused striatal fiber tracts showed mainly regulations in proteins related to neuronal functions. The results were ambivalent regarding functional and cognitive effects. On the one hand, we observed protein regulations that would have unfavorable effects: upregulation of Sncg is associated with neurodegenerative diseases (29), and high circulating levels of Penk have been established as a marker for vascular dementia (30). We observed a reduction of Cstb, Jam3, Zc3h14, and Msn, which can cause neuroinflammation and epilepsy (Cstb) (31), blood-brain-barrier disruption (Jam3) (20), impairment of synaptic function (Zc3h14) (32) and long-term memory (Msn) (18). On the other hand, the observed increase in Atpif1 could rescue cells under hypoxic conditions (16). Induced Ppp1r1a can promote long-term potentiation by inhibiting protein phosphatase 1 (PP1), fostering memory and learning (15). Caprin-1 upregulation was verified by immunohistochemistry. This cell-proliferation regulating protein has an additional role in neurons (33). In the brain, Caprin-1—also known as RNG105—is linked to the formation of cytoplasmic stress granules (34). More importantly, it regulates mRNA localization in dendrites and is essential for long-term memory formation via homeostatic AMPA-receptor scaling (35). Interestingly, a recent preprint described STAT-1 and STAT-3—downstream targets of IL-6—as interacting proteins with Caprin-1 detected by immunoprecipitation and proteomics (36).

In the contralateral cortex, we observed ambivalent effects of protein regulations as well. Upregulated proteins almost exclusively have a link to the plasma membrane system or endomembrane system. Camk2n1 can prevent long-term memory loss (22). However, Camk2n1 is an inhibitor of CaMKII, which is necessary for motor learning and long-term potentiation (23). Interestingly, paracrine IL-6 upregulates Copb1 as part of the COPI complex. Bettayeb et al. showed that the COPI complex could significantly reduce amyloid plaques in Alzheimer's disease, which might prevent cognitive decline (25). Downregulated proteins were part of membrane complexes. Reduced activity of Prodh causes cognitive impairment (26). Albeit, Vamp3, and Gat1 were decreased by paracrine IL-6. Inhibition of Vamp3 reduces activation of microglia, potentially preventing cognitive impairments after surgery (27). Gat1 is a GABA transporter localized in axons, nerve terminals, and astrocytes, removing GABA from the synaptic cleft (37). We observed a widespread reduction of Gat1. Therefore, reduction of Gat1 would lead to induced GABAergic transmission. Gat1 heterozygous mice with reduced Gat1 expression showed enhanced learning (38), whereas Gat knockout mice had learning deficits and an ADHS like phenotype (39). This indicates that there is a dosing effect of Gat1 on cognitive functions. Treatment with the anti-epileptic drug tiagabine, a specific Gat1 inhibitor, does not lead to cognitive impairments in therapeutic dosages (40). Indeed, Gat1 inhibition improved cognitive functions in patients who have epilepsy (41). In the setting of cerebral ischemia, Gat1 inhibition was neuroprotective (42–44). and preserved hippocampal neurons (45).

In summary, paracrine IL-6 promotes cerebral remodeling in the early stage after asymptomatic CCA occlusion on a structural level with increased connectivity in motor systems. Changes in the proteome of the hypoperfused striatum and contralateral motor cortex showed ambivalent results. In terms of cognitive functions, some protein regulations we observed might be unfavorable. These regulations might explain the adverse effects of long-term elevated IL-6 on cognition. However, we identified protein targets that have been shown to reduce cognitive impairments in other pathological settings such as epilepsy or Alzheimer's disease or might improve neuronal resilience to hypoperfusion.

The major limitation of this study lies in its exploratory design. Further studies are needed to confirm the effects of the identified targets on the motor function and cognitive outcomes after CCA occlusion. We modeled the effect of paracrine IL-6 in early stages in a mouse model mimicking asymptomatic carotid artery stenosis. Cognitive decline or flexibility in learning tasks months after the onset of CCA occlusion was not investigated since our focus was exploring cerebral remodeling in the short term. Moreover, the effects should be confirmed in aged mice.

Focusing on the targets identified in our study—either by selectively inhibiting adverse regulations or by enhancing beneficial regulations—might establish a novel treatment strategy to improve long-term outcome in the setting of asymptomatic CCA occlusion by fostering cerebral remodeling. Preventing downregulations of for instance Jam3 or induction of Atpif1 might be challenging. More promising would be the development of a Vamp3 inhibitor. However, most promising would beGat1 inhibition using the FDA-proven anti-epileptic drug tiagabine, which should be evaluated in further studies.

## Data Availability Statement

The mass spectrometry proteomics data have been deposited to the ProteomeXchange Consortium via the PRIDE [1] partner repository with the dataset identifier PXD029737.

## Ethics Statement

The animal study was reviewed and approved by Landesamt für Gesundheit und Soziales Berlin.

## Author Contributions

MTCK, ME, UD, CH, and CJH designed this project and wrote the manuscript. MTCK, SPC, MK, SM, JL, JA, and PB-S performed the experiments and analyzed the results. PM contributed essential experimental advice to mass spectrometry. All authors reviewed and approved the final manuscript.

## Funding

This work was supported by the Deutsche Forschungsgemeinschaft (DFG, German Research Foundation) to CJH (HO5277/3-1) and CH (HA5741/5-1) and Project-ID 424778381 –TRR 295 to CH and ME; Einstein Centre of Regenerative Therapies (Einstein Kickbox starting grant to CJH); and German Federal Ministry of Education and Research (BMBF CSB 01EO1301) to CH and ME. CJH was a participant in the Charité Clinical Scientist Program funded by the Charité-Universitätsmedizin Berlin and the Berlin Institute of Health. MKu received funding from the Graduate School 203 of the DFG Excellence Initiative, Berlin-Brandenburg School for Regenerative Therapies. ME received funding from DFG under Germany's Excellence Strategy – EXC-2049 – 390688087, BMBF, DZNE, DZHK, EU, Corona Foundation, and Fondation Leducq. Funding to SM, SK, and PB-S was provided by the BMBF under the ERA-NET NEURON scheme (01EW1811) and the DFG (BO 4484/2-1). Noninvasive MRI measurements allowed a longitudinal study design and were supported by Charité 3R, Replace – Reduce – Refine.

## Conflict of Interest

The authors declare that the research was conducted in the absence of any commercial or financial relationships that could be construed as a potential conflict of interest.

## Publisher's Note

All claims expressed in this article are solely those of the authors and do not necessarily represent those of their affiliated organizations, or those of the publisher, the editors and the reviewers. Any product that may be evaluated in this article, or claim that may be made by its manufacturer, is not guaranteed or endorsed by the publisher.

## References

[1] Song P Fang Z Wang H Cai Y Rahimi K Zhu Y. Global and regional prevalence, burden, and risk factors for carotid atherosclerosis: a systematic review, meta-analysis, and modelling study. Lancet Glob Health. (2020) 8:e721–9. 10.1016/S2214-109X(20)30117-0 32353319

[2] Howard DPJ Gaziano L Rothwell PM Oxford Vascular S. Risk of stroke in relation to degree of asymptomatic carotid stenosis: a population-based cohort study, systematic review, and meta-analysis. Lancet Neurol. (2021) 20:193–202. 10.1016/S1474-4422(20)30484-1 33609477 PMC7889579

[3] Lal BK Dux MC Sikdar S Goldstein C Khan AA Yokemick J. Asymptomatic carotid stenosis is associated with cognitive impairment. J Vasc Surg. (2017) 66:1083–92. 10.1016/j.jvs.2017.04.038 28712815

[4] Gertz K Kronenberg G Kalin RE Baldinger T Werner C Balkaya M. Essential role of interleukin-6 in post-stroke angiogenesis. Brain. (2012) 135:1964–80. 10.1093/brain/aws075 22492561 PMC3359750

[5] Hoffmann CJ Harms U Rex A Szulzewsky F Wolf SA Grittner U. Vascular signal transducer and activator of transcription-3 promotes angiogenesis and neuroplasticity long-term after stroke. Circulation. (2015) 131:1772–82. 10.1161/CIRCULATIONAHA.114.013003 25794850

[6] Kossmann T Hans V Imhof HG Trentz O Morganti-Kossmann MC. Interleukin-6 released in human cerebrospinal fluid following traumatic brain injury may trigger nerve growth factor production in astrocytes. Brain Res. (1996) 713:143–52. 10.1016/0006-8993(95)01501-9 8724985

[7] Wright CB Sacco RL Rundek T Delman J Rabbani L Elkind M. Interleukin-6 is associated with cognitive function: the Northern Manhattan Study. J Stroke Cerebrovasc Dis. (2006) 15:34–8. 10.1016/j.jstrokecerebrovasdis.2005.08.009 16501663 PMC1382058

[8] Yoshizaki K Adachi K Kataoka S Watanabe A Tabira T Takahashi K. Chronic cerebral hypoperfusion induced by right unilateral common carotid artery occlusion causes delayed white matter lesions and cognitive impairment in adult mice. Exp Neurol. (2008) 210:585–91. 10.1016/j.expneurol.2007.12.005 18222425

[9] Nishino A Tajima Y Takuwa H Masamoto K Taniguchi J Wakizaka H. Long-term effects of cerebral hypoperfusion on neural density and function using misery perfusion animal model. Sci Rep. (2016) 6:25072. 10.1038/srep25072 27116932 PMC4846861

[10] Campbell IL Abraham CR Masliah E Kemper P Inglis JD Oldstone MB. Neurologic disease induced in transgenic mice by cerebral overexpression of interleukin 6. Proc Natl Acad Sci USA. (1993) 90:10061–5. 10.1073/pnas.90.21.10061 7694279 PMC47713

[11] Gyengesi E Rangel A Ullah F Liang H Niedermayer G Asgarov R. Chronic microglial activation in the GFAP-IL6 mouse contributes to age-dependent cerebellar volume loss and impairment in motor function. Front Neurosci. (2019) 13:303. 10.3389/fnins.2019.00303 31001075 PMC6456818

[12] Chesworth R Gamage R Ullah F Sonego S Millington C Fernandez A. Spatial memory and microglia activation in a mouse model of chronic neuroinflammation and the anti-inflammatory effects of apigenin. Front Neurosci. (2021) 15:699329. 10.3389/fnins.2021.699329 34393713 PMC8363202

[13] Emmrich JV Neher JJ Boehm-Sturm P Endres M Dirnagl U Harms C. Stage 1 registered report: effect of deficient phagocytosis on neuronal survival and neurological outcome after temporary middle cerebral artery occlusion (tMCAo). F1000Res. (2017) 6:1827. 10.12688/f1000research.12537.2 29152223 PMC5664978

[14] Jochner MCE An J Lattig-Tunnemann G Kirchner M Dagane A Dittmar G. Unique properties of PTEN-L contribute to neuroprotection in response to ischemic-like stress. Sci Rep. (2019) 9:3183. 10.1038/s41598-019-39438-1 30816308 PMC6395706

[15] Munton RP Vizi S Mansuy IM. The role of protein phosphatase-1 in the modulation of synaptic and structural plasticity. FEBS Lett. (2004) 567:121–8. 10.1016/j.febslet.2004.03.121 15165904

[16] Garcia-Bermudez J Cuezva JM. The ATPase Inhibitory Factor 1 (IF1): A master regulator of energy metabolism and of cell survival. Biochim Biophys Acta. (2016) 1857:1167–82. 10.1016/j.bbabio.2016.02.004 26876430

[17] O'Brien A Marshall CR Blaser S Ray PN Yoon G. Severe neurodegeneration, progressive cerebral volume loss and diffuse hypomyelination associated with a homozygous frameshift mutation in CSTB. Eur J Hum Genet. (2017) 25:775–8. 10.1038/ejhg.2017.39 28378817 PMC5477367

[18] Freymuth PS Fitzsimons HL. The ERM protein Moesin is essential for neuronal morphogenesis and long-term memory in Drosophila. Mol Brain. (2017) 10:41. 10.1186/s13041-017-0322-y 28851405 PMC5576258

[19] Guthrie CR Greenup L Leverenz JB Kraemer BC. MSUT2 is a determinant of susceptibility to tau neurotoxicity. Hum Mol Genet. (2011) 20:1989–99. 10.1093/hmg/ddr079 21355046 PMC3080609

[20] Mochida GH Ganesh VS Felie JM Gleason D Hill RS Clapham KR. A homozygous mutation in the tight-junction protein JAM3 causes hemorrhagic destruction of the brain, subependymal calcification, and congenital cataracts. Am J Hum Genet. (2010) 87:882–9. 10.1016/j.ajhg.2010.10.026 21109224 PMC2997371

[21] Dosemeci A Loo HK Toy D Winters CA Reese TS Tao-Cheng JH. FAM81A protein, a novel component of the postsynaptic density in adult brain. Neurosci Lett. (2019) 699:122–6. 10.1016/j.neulet.2019.02.003 30735723 PMC6443461

[22] Vigil FA Mizuno K Lucchesi W Valls-Comamala V Giese KP. Prevention of long-term memory loss after retrieval by an endogenous CaMKII inhibitor. Sci Rep. (2017) 7:4040. 10.1038/s41598-017-04355-8 28642476 PMC5481336

[23] Wang Q Yin P Yu B Zhao Z Richter-Levin G Yu L. Down-regulation of dorsal striatal alphaCaMKII causes striatum-related cognitive and synaptic disorders. Exp Neurol. (2017) 298:112–21. 10.1016/j.expneurol.2017.09.004 28890075

[24] Van Bergen NJ Ahmed SM Collins F Cowley M Vetro A Dale RC. Mutations in the exocyst component EXOC2 cause severe defects in human brain development. J Exp Med. (2020) 217:e20192040. 10.1084/jem.20192040 32639540 PMC7537385

[25] Bettayeb K Hooli BV Parrado AR Randolph L Varotsis D Aryal S. Relevance of the COPI complex for Alzheimer's disease progression in vivo. Proc Natl Acad Sci USA. (2016) 113:5418–23. 10.1073/pnas.1604176113 27114526 PMC4868486

[26] Raux G Bumsel E Hecketsweiler B van Amelsvoort T Zinkstok J Manouvrier-Hanu S. Involvement of hyperprolinemia in cognitive and psychiatric features of the 22q11 deletion syndrome. Hum Mol Genet. (2007) 16:83–91. 10.1093/hmg/ddl443 17135275

[27] Chen Y Sun JX Chen WK Wu GC Wang YQ Zhu KY. miR-124/VAMP3 is a novel therapeutic target for mitigation of surgical trauma-induced microglial activation. Signal Transduct Target Ther. (2019) 4:27. 10.1038/s41392-019-0061-x 31637007 PMC6799846

[28] Benarroch EE. Periaqueductal gray: an interface for behavioral control. Neurology. (2012) 78:210–7. 10.1212/WNL.0b013e31823fcdee 22249496

[29] Bras J Gibbons E Guerreiro R. Genetics of synucleins in neurodegenerative diseases. Acta Neuropathol. (2021) 141:471–90. 10.1007/s00401-020-02202-1 32740728

[30] Holm H Nagga K Nilsson ED Ricci F Melander O Hansson O. High circulating levels of midregional proenkephalin A predict vascular dementia: a population-based prospective study. Sci Rep. (2020) 10:8027. 10.1038/s41598-020-64998-y 32415209 PMC7229155

[31] Okuneva O Li Z Korber I Tegelberg S Joensuu T Tian L. Brain inflammation is accompanied by peripheral inflammation in Cstb (-/-) mice, a model for progressive myoclonus epilepsy. J Neuroinflammation. (2016) 13:298. 10.1186/s12974-016-0764-7 27894304 PMC5127053

[32] Rha J Jones SK Fidler J Banerjee A Leung SW Morris KJ. The RNA-binding protein, ZC3H14, is required for proper poly(A) tail length control, expression of synaptic proteins, and brain function in mice. Hum Mol Genet. (2017) 26:3663–81. 10.1093/hmg/ddx248 28666327 PMC5886104

[33] Wang B David MD Schrader JW. Absence of caprin-1 results in defects in cellular proliferation. J Immunol. (2005) 175:4274–82. 10.4049/jimmunol.175.7.4274 16177067

[34] Solomon S Xu Y Wang B David MD Schubert P Kennedy D. Distinct structural features of caprin-1 mediate its interaction with G3BP-1 and its induction of phosphorylation of eukaryotic translation initiation factor 2alpha, entry to cytoplasmic stress granules, and selective interaction with a subset of mRNAs. Mol Cell Biol. (2007) 27:2324–42. 10.1128/MCB.02300-06 17210633 PMC1820512

[35] Nakayama K Ohashi R Shinoda Y Yamazaki M Abe M Fujikawa A. RNG105/caprin1, an RNA granule protein for dendritic mRNA localization, is essential for long-term memory formation. Elife. (2017) 6:e29677. 10.7554/eLife.29677 29157358 PMC5697933

[36] Vu L Ghosh A Tran C Tebung WA Sidibe H Garcia-Mansfield K. Defining the caprin-1 interactome in unstressed and stressed conditions. J Proteome Res. (2021) 20:3165–78. 10.1021/acs.jproteome.1c00016 33939924 PMC9083243

[37] Borden LA. GABA transporter heterogeneity: pharmacology and cellular localization. Neurochem Int. (1996) 29:335–56. 10.1016/0197-0186(95)00158-1 8939442

[38] Shi J Cai Y Liu G Gong N Liu Z Xu T. Enhanced learning and memory in GAT1 heterozygous mice. Acta Biochim Biophys Sin. (2012) 44:359–66. 10.1093/abbs/gms005 22318715

[39] Yang P Cai G Cai Y Fei J Liu G. Gamma aminobutyric acid transporter subtype 1 gene knockout mice: a new model for attention deficit/hyperactivity disorder. Acta Biochim Biophys Sin. (2013) 45:578–85. 10.1093/abbs/gmt043 23656791

[40] Aikia M Jutila L Salmenpera T Mervaala E Kalviainen R. Comparison of the cognitive effects of tiagabine and carbamazepine as monotherapy in newly diagnosed adult patients with partial epilepsy: pooled analysis of two long-term, randomized, follow-up studies. Epilepsia. (2006) 47:1121–7. 10.1111/j.1528-1167.2006.00545.x 16886974

[41] Dodrill CB Arnett JL Shu V Pixton GC Lenz GT Sommerville KW. Effects of tiagabine monotherapy on abilities, adjustment, and mood. Epilepsia. (1998) 39:33–42. 10.1111/j.1528-1157.1998.tb01271.x 9578010

[42] Chen Xu W Yi Y Qiu L Shuaib A. Neuroprotective activity of tiagabine in a focal embolic model of cerebral ischemia. Brain Res. (2000) 874:75–7. 10.1016/S0006-8993(00)02554-3 10936225

[43] Yang Y Li Q Wang CX Jeerakathil T Shuaib A. Dose-dependent neuroprotection with tiagabine in a focal cerebral ischemia model in rat. Neuroreport. (2000) 11:2307–11. 10.1097/00001756-200007140-00048 10923691

[44] Lie ME Gowing EK Clausen RP Wellendorph P Clarkson AN. Inhibition of GABA transporters fails to afford significant protection following focal cerebral ischemia. J Cereb Blood Flow Metab. (2018) 38:166–73. 10.1177/0271678X17743669 29148909 PMC5757447

[45] Inglefield JR Perry JM Schwartz RD. Postischemic inhibition of GABA reuptake by tiagabine slows neuronal death in the gerbil hippocampus. Hippocampus. (1995) 5:460–8. 10.1002/hipo.450050508 8773258

