# Supplementary material for: Paracrine Interleukin 6 Induces Cerebral Remodeling at Early Stages After Unilateral Common Carotid Artery Occlusion in Mice
Source: Front Cardiovasc Med. 2022 Jan 27;8:805095. doi: 10.3389/fcvm.2021.805095 (PMC8830347; doi:10.3389/fcvm.2021.805095)
Supplement: Supplementary file 1 [file Data_Sheet_1.docx]

Supplementary Material

# Supplementary Data

**Expanded Materials and Methods**

Animals

All experimental procedures were approved by the local authorities (Landesamt für Gesundheit und Soziales, LaGeSo), Berlin (Reg G0119/16)) and were conducted in accordance with the German animal protection law and local animal welfare guidelines. Mice are group-housed with *ad libitum* access to water. For staircase testing mice had restricted access to food limited to 3 h per day as a motivator for the behavioral task. Weight and temperature of the mice are recorded daily, and an additional chow pellet of 1.1 g is given for the food-restricted period. One day before and after surgery, animals are allowed *ad libitum* access to food. Cages are equipped with environmental enrichment (red transparent plastic nest box and brown paper towels). Mice are kept in specific pathogen free conditions under a 12 h light/dark cycle at 22 ± 1 °C.

We genetically engineered a mouse model by inducing a FLEX cassette into the ROSA26 locus containing an inverted IL-6 followed by a T2A self-cleavage sequence and mKate2. IL-6 was fused to a C-terminal myc-tag (EQKLISEEDL) via a spacer (GGSGGTGGS). Mice were crossbred with astrocyte specific Cx30-Cre-ERT2 mice. The mice were bred locally on a C57BL/6J background and were backcrossed for 10 generations. We used mice for experiments which where hemizygous for Cx30-Cre-ERT2 and hemizygous for FLEX-IL-6. Mice which were hemizygous for FLEX-IL-6 served as control animals. The mice were at an age of 10 – 12 weeks. In total, 66 mice were used for the experiments.

Mouse groups were age- and sex-matched for experimental and control groups. Mice were excluded determined by a priori definition if they had visible stroke in MRI imaging 24 h after surgery. Experimenters were blinded during behavioral assessment, tissue processing and data analysis.

Western Blot

For brain IL-6 analysis using western blot at day 7 after the first tamoxifen injection, 4 C57BL/6J, 4 FLEX-IL6, and 6 Cx30-Cre-ERT2;FLEX-IL6 mice were used. Deep frozen mouse brain tissue was lysed in lysis buffer containing 20 mM Tris (pH 7.5), 150 mM NaCl, 2 mM EDTA, 2 mM MgCl2, 10% glycerol, protease and phosphatase inhibitor cocktails (Halt™, ThermoScientific), on a rotor stator tissue homogenizer for 2 min at 18000 rpm while kept on ice. The lysates were kept on ice for 20 min. and centrifuged (10000xg, 10 min.). 0.5% Triton-X and 0.5% NP40 were added to the supernatant and the mixture rotated at 4°C for 1 h. The samples were then centrifuged at 10000xg, 4°C for 15 min and the supernatant was collected. Samples were then sonicated for 10 s at 10% power, reduced with NuPage LDS sample buffer and NuPage Reducing Agent (10x) and denatured at 70°C for 10 min. Samples were loaded on precast 4-12% gels (Life Technologies) and run for 35 min at 200 V in MES buffer (#NP0002, Novex life technologies). Western Blot was performed on a wet-blot for one hour at 30 V onto a nitrocellulose membrane. Proteins were fixed in PBS/0.4% formaldehyde and washed with Ready Tector wash buffer. Antibody incubation was done in Ready Tector HRP-solution (mouse/rabbit) for 1.5 h at room temperature. After washing, bands were imaged using WesternBright ECL HRP substrate (Advansta) and imager (Vilber Lourmat).

Enzyme-linked immunosorbent assay

Thirteen FLEX-IL6, 9 Cx30-Cre-ERT2;FLEX-IL6 and 7 VeCdh-Cre-ERT2;FLEX-IL6 mice were used for serum IL‑6 and white blood cell (WBC) analysis. Due to low readout quality, 2 VeCdh-Cre-ERT2;FLEX-IL6 mice were excluded for WBC analysis and 1 FLEX-IL6 mouse was excluded for serum IL-6 analysis due to low quantity of serum. Serum was thawed and IL-6 concentration determined by ELISA measurements (Mouse IL-6 Quantikine, RnD Systems). Measurements were normalized to protein concentration measured using BCA assay (Pierce reagent, Thermo Fisher).

Cell culture

HEK cells were purchased from Biocat GmbH (Heidelberg, Germany) and cultured in DMEM (DMEM (Gibco) supplemented with 10% fetal calf serum, 1% penicillin/streptomycin (Merck), 1% sodium pyruvate(Gibco), 1% MEM non-essential amino acids (Gibco) and 1% GlutaMAX (Gibco). Brain microvascular endothelial cells (bEnd.3, ATCC CRL-2299) were purchased from ATCC and cultured in DMEM (ATCC) supplemented with 10% FCS, L-glutamine and antibiotics. HEK cells were transfected with plasmids containing the FLEX-IL6 construct and/or a plasmid containing Cre-EGFP using XtremeGene (Roche). The cell culture supernatant was collected 24 h after transfection and centrifuged to remove cellular debris. Endothelial bEnd.3 cells were incubated with the conditioned cell culture supernatant from Cre-transfected or Cre- and FLEX-IL6 co-transfected HEK cells for 1 h.

Real-time RT-PCR

Real-time RT-PCR was performed as described (Hoffmann et al., 2015). In brief, RNA was extracted using TRIzol (Invitrogen) and stored at -80 °C prior to transcription. One microgram of RNA was transcribed with MLV reverse transcriptase (Promega) using random primers (Roche) and oligo-dT primers (Eurofins-MWG). Real-time PCR was performed with exon-spanning primers for tyrosine 3-monooxygenase/tryptophan 5-monooxygenase activation protein (Ywhaz) and intron-spanning primers for Il6 using SYBR Green (Qiagen). For Primer Sequences, see Supplemental Table 1. Comparable primer efficiency was confirmed. Melting curves were analyzed for all runs. Experiments in the absence of template and those with untranscribed RNA served as negative controls. Fold differences in mRNA levels compared to internal control levels were calculated using the ddCT method.

Mouse Surgery

Mice were subjected to a unilateral ligation of the left common carotid artery. Following the 3R principle of animal experiment reduction, mice with unilateral CCA occlusion served additionally as sham control for a tMCAO experiment and surgery was performed analogous to the sham Operation as established in our lab. Mice were anaesthetized with 1.5-3.5% Isoflurane and maintained in 1.0-2.5% Isoflurane with approximately 75/25 N_2_O/O_2_. Body temperature was maintained at 37.0 +/- 0.5 °C using a heating plate or homoeothermic blanket system. A midline neck incision was made and the soft tissues are pulled apart. The left common carotid artery (CCA) was carefully dissected free from the surrounding nerves (without harming the vagus nerve) and a ligature is made using suture thread. Then the external carotid artery (ECA) on the same side was separated and a second knot was made. Next, the internal carotid artery (ICA) was isolated, and a loose knot prepared with a suture thread. Both the ICA and the pterygopalatine artery (PA) were clipped. A small hole was cut in the CCA before it bifurcates to the ECA and the ICA. A disinfected Doccol® filament or a monofilament made of 8/0 nylon coated with silicon hardener mixture was then introduced into the artery and immediately withdrawn. The remaining threads were cut and the CCA occlusion maintained. For pain relief, bupivacain gel (1%) was topically applied to the wound, and the wound was closed with an adaptive suture. Following surgery, the animals had access to soaked and mashed chow in petri dishes for up to 24 hours. On day 2 after surgery, animals received i.p. injections of 1 mg tamoxifen (Sigma, 10 mg/ml in 1:10 ethanol/corn oil) for one to three consecutive days.

Behavioral Tests

For behavioral experiments and DTI analysis with 21 days survival after surgery, 15 mice (8 FLEX-IL6, 7 Cx30-Cre-ERT2;FLEX-IL6) were used. According to preset exclusion criteria, 1 Cx30-Cre-ERT2;FLEX-IL6 and 1 FLEX-IL6 mouse were excluded due to ischemic lesions seen in MRI imaging at day 1. Sickness behavior was assessed by modified DeSimoni’s neuroscore at days 2, 7, 14 and 21 following unilateral CCA occlusion. The maximum score is 43 points, where a higher score indicates more deficits. Rotarod test was performed with three runs per trial with a recovery time of 30 min between trials. Shown is the average performance. Tests were performed on days 2, 7, and 14 after unilateral CCAo. Staircase skilled pellet reaching test is performed daily for 21 days of training before and up to 21 days after ligation of the CCA for conditioning as described previously (Emmrich et al., 2017). Outcome is measured as relative performance compared to the individual performance before surgery for each forepaw.

Magnetic Resonance Imaging

During the examination animals were placed on a heated circulating water blanket to ensure constant body temperature of 37 °C. Anaesthesia was induced with 2.5 % and maintained with 2.0 – 1.5 % isoflurane (Forene, Abbot, Wiesbaden, Germany) delivered in a O2 / N2O mixture (0.3 / 0.7 l/min) via a facemask under constant ventilation monitoring (Small Animal Monitoring & Gating System, SA Instruments, Stony Brook, New York, USA). Animals underwent in vivo magnetic resonance imaging (MRI) at day 1 post CCA occlusion at a 7 T horizontal rodent scanner (Bruker PharmaScan, Bruker, Ettlingen, Germany) and Paravision 5.1 software using a 20mm diameter Tx/Rx quadrature volume resonator (Rapid Biomedical, Rimpar, Germany). Morphometric T2-weighted (T2w) images were acquired with a 2D-RARE sequence with repetition time TR/echo time TE=4200 ms/36 ms, RARE factor 8, 4 averages, 32 axial slices with a slice thickness of 0.5 mm, field of view FOV = 25.6 x 25.6 mm^2^, matrix size of 256x192 (zerofilled to 256x256), Bandwidth BW=46.9 kHz and total acquisition time TA=6:43 min. T2w and diffusion tensor imaging (DTI) at 21 d post surgery were carried out at a 7 T Bruker BioSpec with a TX/RX 1H-cryoprobe and ParaVision 6.0.1 software. For T2w MRI at 21 d a 2D-RARE sequence with TR/TE = 4250 ms / 33 ms, RARE factor 8, 2 averages, 40 axial slices with a slice thickness of 0.4 mm, FOV=19.2 x 19.2 mm^2^, matrix of 192 x 192, BW=34.7 kHz and TA=3:24 min was used. DTI was performed with a single shell (60 diffusion directions at b=1000 s/mm2, 5 b=0 images) 2D segmented spin echo EPI sequence (4 segments) with matching slice thickness and FOV but lower resolution (matrix=160x160, TR/TE=3500 ms/TE=30 ms, BW=300 kHz, diffusion gradient duration/separation= 2.7 ms/8.6 ms, TA=15:10 min).

Diffusion tensor imaging analysis

All MR images were converted to NIFTI format, T2w images were segmented into tissue probability maps of gray/white matter and cerebrospinal fluid and a custom brain atlas with 308 anatomical regions (154 right/154 left hemisphere) derived from the Allen mouse brain atlas was registered to the T2w using ANTx2 (<https://github.com/ChariteExpMri/antx2>). Image coregistration of T2w images and matching atlases to b=0 images allowed atlas-based connectivity analysis of DTI data. Connectomes were generated in mrtrix (<https://www.mrtrix.org>). The processing pipeline is openly available (https://github.com/ChariteExpMri/rodentDtiConnectomics) and consists of

1. Denoising, Gibbs ringing removal, bias field correction, eddy current correction and motion correction
2. Diffusion orientation function reconstruction using constrained spherical deconvolution
3. Connectome reconstruction using streamline tractography and SIFT2 optimization
4. Reconstruction of connectivity matrices counting the number of streamlines from atlas region to region

Exploratory group statistical comparisons were made for each matrix entry (i.e. number of streamlines between pairs of regions) using t-tests and threshold p value of 0.001 and visualized in custom MATLAB tools (MathWorks, Natick, MA, USA).

Proteomic analysis of LCM (Laser Capture Microscopy) samples by mass spectrometry

For the proteomics experiments at day 5 after surgery, 9 (4 FLEX-IL6, 5 Cx30-Cre-ERT2;FLEX-IL6) mice were used . Frozen tissue was sectioned using a Cryostat into 20 µm thin sections and mounted on PEN membrane slides (ThermoFisher Scientific). After drying at RT, tissue was fixed for 10 min in 1:1 methanol/acetone solution, left to dry and then stored on dry ice until further processing. Tissue sections were cut using a laser capture microscope (Arcturus XT LCM instrument, Thermo Fisher Scientific) by selecting the desired areas in the ipsilateral striatum and the contralateral motor cortex. Dissected areas were collected in 30µl 6M Guanidinium Hydrochloride buffer and heated for 10minutes at 95°C. Samples were reduced in 1mM DTT for 30min and alkylated in 5mM Chloroacetamide for 30min. The samples were first digested using 0.5µg endopeptidase LysC (Wako, Osaka, Japan) for 4 hours, followed by a 1:4 dilution in 50mM ammonium bicarbonate (pH = 8.5) and digestion with 1µg sequence-grade trypsin (Promega) for 16h. The digestion was stopped by acidifying each sample to pH < 2.5 with 10% trifluoroacetic acid solution. Resulting peptides were purified and stored on stage tips.

LC-MS/MS analyses was performed as described in (Jochner et al., 2019). Peptides were eluted using Buffer B (80% Acetonitrile and 0.1% formic acid) and organic solvent was evaporated using a speedvac (Eppendorf). Peptide samples were resolved in Buffer A (3% acetonitrile and 0.1% formic acid) and separated on a reversed-phase column (20 cm fritless silica microcolumns with an inner diameter of 75 µm, packed with ReproSil-Pur C18-AQ 1.9 µm resin (Dr. Maisch GmbH)) using a 90 min gradient with a 250 nl/min flow rate of increasing Buffer B concentration (from 2% to 60%) on a High Performance Liquid Chromatography (HPLC) system (ThermoScientific). Peptides were ionized using an electrospray ionization (ESI) source (ThermoScientific) and analyzed on a Thermo Orbitrap Fusion (Q-OT-qIT, Thermo). The mass spectrometer was run in data dependent mode selecting the top 20 most intense ions in the MS full scans, selecting ions from 350 to 2000 m/z, using 60K resolution with a 4 × 105 ion count target and 50 ms injection time. Tandem MS was performed by quadrupole isolation at 0.7 m/z, HCD fragmentation with normalized collision energy of 32 and resolution of 15K. The MS2 ion count target was set to 5x104 with a maximum injection time of 250 ms. Only precursors with charge state 2–7 were sampled for MS2. The dynamic exclusion duration was set to 30 s with a 10 ppm tolerance around the selected precursor and its isotopes.

Data were analyzed using MaxQuant software package (v1.6.0.1) and a decoy mouse UniProt database (MOUSE.2017-01) containing forward and reverse sequences. The search included variable modifications of methionine oxidation and N-terminal acetylation, deamidation (N and Q) and fixed modification of carbamidomethyl cysteine. Minimal peptide length was set to seven amino acids and a maximum of 3 missed cleavages was allowed. The FDR (false discovery rate) was set to 1% for peptide and protein identifications. Unique and razor peptides were considered for quantification. MS2 identifications were transferred between runs with the “Match between runs” option. Protein intensities were normalized using the in-built LFQ (label-free quantification) algorithm. Output data were filtered to exclude reverse database hits and potential contaminants. Statistical data analysis was performed using Perseus software (v1.6.2.1). Biological replicates for each condition were defined as groups and intensity values were filtered for “minimum value of 3” in at least one group. After log2 transformation missing values were imputed with random noise simulating the detection limit of the mass spectrometer. Differences in protein abundance between the experimental groups were calculated using two-sample Student´s t test. Significance cut-off was set to p-value ≤ 0.05 and fold change > 2/< -2. )

After tissue processing and proteome analysis, we excluded one wild-type and one experimental animal due to technical reasons. In both animals, the amount of sampled tissue was too low for further analysis.

Histology

Mice were euthanized for histological and biochemical analyses 5 days after unilateral CCA occlusion. A dose of 100 µl Ketamine/Xylazine (0.7% Ketamine (10%, cb pharma), 0.8 % Xylazine (20 mg/ml Xylavet, cb pharma)) per 10 g body weight was given i.p.. Mice were placed on a 35°C heating pad until they reached deep anesthesia and showed no pain reflex on the front paw. Then, a lateral abdominal incision was made and 500 µl of blood sampled from the vena cava and analyzed with scil Vet abc animal blood counter (scil animal care company, Viernheim, Germany; mouse program). The mice were then perfused transcardially with 0.9% saline water. For proteome analysis, protease inhibitor was added (1 tablet/50 ml saline solution) (cOmplete™, Roche). The brains were removed and deep frozen in dry-ice cooled 2‑methylbutane. Blood was left to coagulate for 30 min at RT, centrifuged at 4°C at 3800 rpm and the serum transferred to a new tube. Tissue and serum were stored at -80 C until further processing.

For the proteomics histology assessment of GAT-1 with 5 day survival, 12 (6 FLEX-IL6, 6 Cx30-Cre-ERT2;FLEX-IL6) mice were used. For all histological preparations, tissue was cut in 20 µm sections and mounted on slides. Sections were fixed for 10 min in -20°C 1:1 acetone/methanol. Sections were rehydrated in PBS and blocked for 1 h in PBS with 10% NDS and 0.01% Triton-X100 (Sigma). Antibodies were diluted in the blocking buffer and incubated over night at 4°C. For antibody details, please see the Major Resources Table in the Supplemental Materials. Nuclei were stained with DAPI (4′,6-Diamidin-2-phenylindol) 1:2000 diluted in PBS. To reduce endogenous background fluorescence, sections were incubated for 10 min with 0.3% Sudan black dissolved in 70% ethanol. Imaging was done on a Leica SP8 confocal microscope using LAS X software on a 20x objective (HC PL APO CS2).

Image analysis

Cell quantification was done with ImageJ and CellProfiler software on image areas representative of the area chosen for laser capture microdissection (LCM) and as indicated in the figures.

Methods to prevent bias

The experimenters were blinded to all experiments, which required manual rating. Experimenters were blinded to the genotype. Reporting conforms to the ARRIVE guidelines.

Statistical analyses

All data are presented as scatter dot plots with the mean ± standard deviation. Data were analyzed with GraphPad Prism version 8.2.0. A detailed description of the corresponding statistical analysis is provided in the figure legends.

References

Emmrich, J.V., Neher, J.J., Boehm-Sturm, P., Endres, M., Dirnagl, U., and Harms, C. (2017). Stage 1 Registered Report: Effect of deficient phagocytosis on neuronal survival and neurological outcome after temporary middle cerebral artery occlusion (tMCAo). *F1000Res* 6**,** 1827. doi: 10.12688/f1000research.12537.3.

Hoffmann, C.J., Harms, U., Rex, A., Szulzewsky, F., Wolf, S.A., Grittner, U., et al. (2015). Vascular signal transducer and activator of transcription-3 promotes angiogenesis and neuroplasticity long-term after stroke. *Circulation* 131(20)**,** 1772-1782. doi: 10.1161/CIRCULATIONAHA.114.013003.

Jochner, M.C.E., An, J., Lattig-Tunnemann, G., Kirchner, M., Dagane, A., Dittmar, G., et al. (2019). Unique properties of PTEN-L contribute to neuroprotection in response to ischemic-like stress. *Sci Rep* 9(1)**,** 3183. doi: 10.1038/s41598-019-39438-1.

**
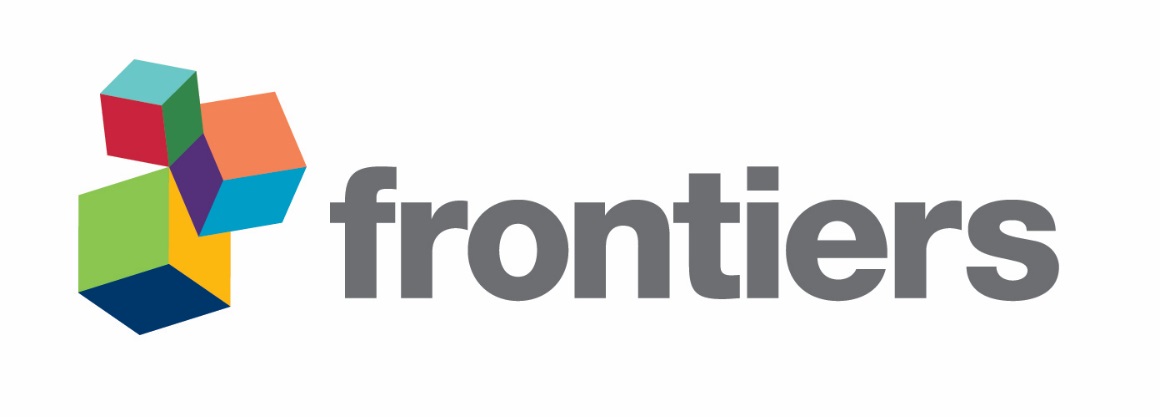
**
